# Long-Term Effects of Early Low-Phosphorous Nutritional Conditioning on Broiler Chicken Performance, Bone Mineralization, and Gut Health Under Adequate or Phosphorous-Deficient Diets

**DOI:** 10.3390/ani14223218

**Published:** 2024-11-09

**Authors:** Núria Tous, Maria Francesch, Joan Tarradas, Ignacio Badiola, Ana M. Pérez de Rozas, Emma Fàbrega, Maria Ballester, Raquel Quintanilla, David Torrallardona

**Affiliations:** 1 IRTA, Animal Nutrition, Mas Bové, 43120 Constantí, Catalonia, Spain; nuria.tous@irta.cat (N.T.); maria.francesch@irta.cat (M.F.); joan.tarradas@irta.cat (J.T.); 2 IRTA, Animal Health, Centre de Recerca en Sanitat Animal (CReSA), Campus de la Universitat Autónoma de Barcelona (UAB), 08193 Bellaterra, Catalonia, Spain; ignacio.badiola@irta.cat (I.B.); ana.perezderozas@irta.cat (A.M.P.d.R.); 3 IRTA, Animal Welfare, Finca Camps i Armet, 17121 Monells, Catalonia, Spain; emma.fabrega@irta.cat; 4 IRTA, Animal Breeding and Genetics, Torre Marimon, 08140 Caldes de Montbui, Catalonia, Spain; maria.ballester@irta.cat (M.B.); raquel.quintanilla@irta.cat (R.Q.)

**Keywords:** chicken, phosphorous restriction, calcium and phosphorus digestibility, phosphorous efficiency, bone mineralization

## Abstract

Phosphorous is essential for many metabolic functions and the constitution of bones. Poultry have limited ability to use phosphorous from diets, which is mainly excreted and causes environmental concern. For this reason, diets are commonly supplemented with inorganic phosphorus and phytases. It has been suggested that chickens can adapt to an early nutrient restriction by increasing its efficiency of utilization, an adaptation that has been defined as nutritional conditioning. The aim of this study was to investigate a strategy of phosphorous nutritional conditioning by feeding low phosphorous diets during the first week of life as a strategy to improve the efficiency of phosphorous utilization later in life. To test this, 600 male broiler chickens were fed either a standard (control group) or a phosphorous-deficient diet (conditioned group) during the first week of life. Later in life, the effect of conditioning was tested using standard or P-deficient diets during the finishing phase (21–43 d). Conditioning did not affect overall performance, despite finding evidence for reduced relative phosphorous excretion between days 19 to 21, increased duodenal gene expression for the phosphorous transporter *SLC34A2* at day 30 (−6% and +17%, respectively), and tendencies for improved phosphorous digestibility (+7%) and tibia mineralization (+6%) at the end of the trial. It is concluded that phosphorous nutritional conditioning early in life can increase subsequent dietary phosphorous utilization and bone mineralization in poultry, although it may not be able to counteract severe phosphorous deficiencies. Further research is required to assess the extent to which phosphorous supplementation in post-conditioning diets may be reduced with this strategy without compromising performance.

## Simple Summary

Phosphorus is a very important nutrient in poultry feeding, and it is involved in many vital metabolic functions and is also a constituent of bones. Despite this, poultry diets have a very low phosphorous availability, which is commonly compensated with the dietary supplementation with inorganic sources of phosphorous and phytases. This study investigated nutritional conditioning with low-phosphorous diets during the first week of life as a strategy to improve the efficiency of phosphorous utilization in broiler chickens. No effect of conditioning on the overall performance was observed, despite evidence for reduced phosphorous excretion, increased duodenal gene expression for phosphorous transporter *SLC34A2*, and improved phosphorous digestibility and tibia mineralization. It is concluded that phosphorous nutritional conditioning in early life has the potential to increase the dietary phosphorous utilization in poultry, although additional research is required to optimize the duration and extent of the conditioning protocol, as well as to quantify to which extent the dietary supplementation with inorganic phosphorous could be reduced with this strategy.

## 1. Introduction

Phosphorous (P) excretion in manure is one of the major environmental concerns associated with intensive poultry production. Nutritional strategies such as the use of diets with reduced phosphorous concentration, the addition of phytases, and the use of genetically modified feed ingredients with lower phytate-P concentration, have been developed to minimize phosphorous excretion [1].

There are indications that broiler chickens can adapt to an early nutrient restriction by increasing their absorptive capacity for this nutrient at the intestinal level and its efficiency of utilization. Such adaptation, defined as nutritional conditioning, has been described in broiler chickens [2]. However, although these improvements have been observed as short-term responses to the deficiency, it is not clear that these changes persist throughout the whole life [3]. Specifically for phosphorous, it has been shown that chicks receiving P- and calcium (Ca)-deficient diets early in life had better P and Ca absorption and similar or better performance and bone mineralization than birds that had been offered adequate diets during subsequent growing periods in which both groups received phosphorous-deficient diets [2,4,5]. Although chickens respond to early dietary deficiencies in P and Ca with improvements in the digestive efficiency for these nutrients in later phases of life, the level of compensation in terms of growth performance and bone mineralization also depends on the P and Ca levels in the subsequent diet [6,7]. However, the mechanisms implied in this process remain unclear.

Some studies link these improvements with an adaptive process based on the modulation of the expression of certain genes encoding intestinal P and Ca transporters [6,8]. Type IIb Sodium-Phosphate Cotransporter (*SLC34A2*) is considered the major sodium-P cotransporter expressed in the apical membrane of enterocytes (mainly in duodenum), and it is involved in removing inorganic phosphorous from the lumen [5,9,10]. In addition to the changes in intestinal transport, the compensatory mechanism can also be modulated by endocrine regulators of mineral homeostasis such as calcitriol and parathyroid hormone [7].

On the other hand, there is little or no information on the effects that those dietary Ca and P reductions may have on the gastrointestinal tract (GIT) immune response of chicken. It was found that reduced dietary Ca and P levels enhanced cytokine expression in the spleen, with an IL-12/IL-10 ratio in favor of an anti-inflammatory status [11]. In addition, the modulation of proinflammatory cytokines in the intestine by dietary Ca and P levels has also been observed in weaned pigs [12], suggesting that a proper functioning immune system depends on dietary phosphorous availability [13].

We hypothesized that phosphorous nutritional conditioning would induce an adaptative response to improve phosphorous utilization later in life, particularly under conditions of phosphorous restriction. Consequently, the main objective in this study was to investigate phosphorous nutritional conditioning during the first week of life as a strategy to improve the efficiency of phosphorous utilization in broiler chickens being fed phosphorous-adequate or phosphorous-deficient diets later in life, and to evaluate the effects of this strategy on lifetime performance, bone mineralization, *SLC34A2* gene expression, immunological response, and morphometry of intestinal mucosa.

## 2. Materials and Methods

### 2.1. Animals, Housing, and Diets

At the start of the trial, 600 male broiler chickens (Cobb 500 FF) were distributed among 24 floor pens of 2.25 m² (25 chickens/pen). Feed and water were provided ad libitum throughout the experiment. The farm’s standard programs for temperature and lighting were used. The temperature program was adjusted to 32–34 °C for the first 2 days, 29–31 °C between days 3 to 7, 26–28 °C, 23–25 °C, and 20–22 °C during the second, third and fourth week, respectively, and 19–21 °C from week 5 until the end of the trial. The lighting program was 24L:0 D from 1 to 2 d and 18L:6D from 3 to 43 d.

The experimental period consisted of three feeding phases: starter from 1 to 7 d of age, grower from 7 to 21 d of age, and finisher from 22 to 43 d of age. In the starter phase, diets were based on corn and soybean meal and they were formulated to meet or exceed the chicken’s requirements according to the Cobb 500 FF breeder recommendations (Cobb-Vantress, 2013), except for P and Ca, which were either supplied at recommended levels to one half of the chicks (control) or at reduced levels (decreases of 2.4 g/kg of non-phytate P and 3.1 g Ca/kg) to the other half (conditioned). In the grower phase, all the birds received a common experimental diet fulfilling nutrient recommendations. In the finisher phase, the chickens in each conditioning group were further divided into two groups that were either fed a diet with recommended P and Ca levels (P-bal) or a diet with reduced P and Ca levels (P-def). In the P-def diets, decreases of 2.4 g non-phytate P/kg and 3.1 g Ca/kg were also applied. This resulted in a 2 × 2 factorial arrangement of treatments with conditioning (CON) in the starter phase (control or conditioned) and phosphorous level (PL) during the finisher phase (P-bal or P-def) as main factors (Table 1). A constant Ca:total P ratio of 1.3 was maintained for all diets to prevent the negative effects of excessive Ca intake and to ensure that Ca did not limit phosphorous utilization. The composition and nutrient contents of the diets are presented in Table 2. The feeds did not contain any anticoccidial drug, antibiotic growth promoter, nor phytase or other feed additives. Titanium dioxide at 5 g/kg was added on top of the grower and finisher feeds as inert tracer for digestibility determinations.

At day 14, the birds from two of the control pens and two of the conditioned pens (48 chicks from each group) were moved to individual wire cages in a separate room until day 22. Their individual nutrient apparent total tract digestibility (ATTD) and P and N excretion were evaluated between 19 and 21 d of trial. At day 22, 12 chickens from each group were euthanized by carbon dioxide inhalation induced by introducing them into a device designed for this purpose, and tissues were sampled to assess villus height and crypt depth, and to determine the gene expression of *SLC34A2* and selected immunological markers in gut mucosa.

At day 22, the birds from an additional four pens (one pen from each factorial treatment, 24 chicks per pen) were again allocated in individual cages until day 30 to measure individual nutrient ATTD and P and N excretion between 27 and 29 d. Again, at 30 d, 12 chickens from each treatment were euthanized to evaluate villus height and crypt depth, *SLC34A2* gene expression, and immunology. In addition, samples of intestinal contents and tibia bones were obtained to measure P-apparent prececal digestibility (APCD) and bone mineralization, respectively.

Performance, P-APCD, tibia mineralization, and gait score were measured up to day 43 of trial in the group-housed chicks while allocated in pens.

### 2.2. Growth Performance and Gait Score in Group Housed Animals

Chicks were weighed in bulk on arrival, and per pen at 7, 14, 21, and 42 d of trial. Pen ADG, ADFI, and feed conversion ratio (g feed/g gain) (FCR) were calculated for the periods 1–7 d, 7–14 d, 14–21 d, and 21–42 d, and for the overall experiment (1–42 d).

At the start of the trial, none of the birds presented signs of lameness. Gait score was individually evaluated in 112 broilers at 38 d of age (7 chicks per pen; 28 chicks per treatment) according to Kestin et al. [14], by allowing the birds to walk individually for 2 m. Scores ranged from 0 (no detectable abnormality) to 5 (complete lameness).

### 2.3. Apparent Total Tract and Apparent Prececal Digestibility

To measure nutrient ATTD and N and P excretion in the individually housed chickens, titanium dioxide was used as inert tracer. Representative samples of excreta from each chick were collected during three days in each of the two periods considered (19–21 and 27–29 d, respectively) and frozen at −18 °C. Afterwards, excreta were freeze-dried, ground, and stored until analysis. Dry matter was determined according to the AOAC method 925.09 [15]. Gross energy was measured with an adiabatic bomb calorimeter (IKA, C-400, Janke & Kunkel KG., Staufen I. Br., Germany; DIN 51900). Nitrogen was determined according to the Dumas AOAC method 968.06, with a nitrogen/protein analyzer FP-528 (LECO Instrumentos, Spain) [15]. Total phosphorous was determined calorimetrically by the vanadomolybdate method 965.17 [15], and the concentrations of TiO_2_ were measured as described by Short et al. [16]. For the determination of the ATTD of N, the content of uric acid in the excreta was determined [17] and it was subsequently subtracted from the total N excreted, considering a value of 33.3% of N in uric acid.

Phosphorous APCD at day 30 was determined using the individually housed birds, whereas the chicks kept in pens were used for APCD at d 43. Birds were humanely euthanized; their intestines were dissected, and the ileum section was gently flushed to obtain the ileal contents. The ileum was defined as the segment before the ileo-cecal junction equaling the length of the cecum. At d 30, the ileal digesta samples from individual birds were analyzed (=12 per treatment). At 43 d, pools of ileal digesta from 3 birds (9 chicks and 3 pools per pen) were used, also resulting in 12 composite samples per dietary treatment. Samples were freeze-dried and ground. Titanium dioxide and phosphorous concentrations were analyzed in feeds and ileal contents.

ATTD and APCD were calculated as follows:

Digestibility (%) = (1 – [(Marker_feed_ / Marker_dig or fec_) × (Nut_dig or fec_ / Nut_feed_)]) × 100

where Marker_feed_ = titanium dioxide concentration in feed; Marker_dig or fec_ = titanium dioxide concentration in ileal content or feces; Nut_dig or fec_ = Nutrient concentration in ileal content or feces; Nut_feed_ = Nutrient concentration in feed.

### 2.4. Bone Mineralization

The complete left leg quarter (including femur, tibia, metatarsus, and feet) was removed from the same birds that were used for phosphorous APCD determination at 30 and 43 d (12 chicks per treatment each day), and were stored at −18 °C. The frozen legs were autoclaved, the tibias were dissected, weighed, dried overnight (103 ± 2 °C), and their ash content was determined (550 °C for 72 h).

### 2.5. Cotransporter SLC34A2 Expression

Samples of epithelium in the mid-duodenum and mid-jejunum were also collected from the same birds at days 22 and 30. Total RNA was extracted from 0.05 g of sample using a Ribopure^TM^ RNA Purification kit ref. AM1924 (Invitrogen-Thermo Fisher Scientific, Alcobendas, Spain) according to the manufacturer’s protocol. RNA was quantified using the Nanodrop ND-1000 (Thermo Fisher Scientific, Alcobendas, Spain). Complementary cDNA was synthesized from 1 µg of total RNA using PrimeScript™ RT reagent Kit Perfect Real Time (Takara Bio Europe SAS, Saint-Germain-en-Laye, France), with random 6-mers primers following the manufacturer’s instructions.

Real time PCR reactions were performed with 7500 Fast RT-PCR system (Applied Biosystems-Thermo Fisher Scientific, Alcobendas, Spain) in a total volume of 20 µL PCR mixture containing 10 µL of SYBR® Select Master Mix ref. 4472908 (Applied Biosystems-Thermo Fisher Scientific, Alcobendas, Spain), 0.6 µL of each forward and reverse primer (both 300 nM), and 5 µL of cDNA (1/16 dilution). The primers are detailed in Supplementary Table S1. The PCR conditions were 95 °C for 10 min followed by 40 cycles of 95 °C for 15 s and 60 °C for 1 min. In addition, a melting temperature curve was determined following the thermal cycling protocol to assess the specificity of the reactions. PCR runs for each sample were performed in duplicate. Relative gene expression was measured applying the 2^−ΔΔCT^ method [18] using β2-microglobulin and GAPDH as reference controls and the control/P-bal group as a calibrator.

### 2.6. Morphometry of Intestinal Mucosa and Gut Immunology

Intestinal sections (two-centimeter-long) from the mid-jejunum around Meckel’s diverticulum were obtained from the individually housed birds that were euthanized at day 22 (12 chicks per treatment) and at day 30 (8 chicks per treatment). Tissues were fixed with formalin–PBS, dehydrated and embedded in paraffin, sectioned at 3 μm, and stained with hematoxylin and eosin. The morphometric measurements for villus height (VH) and crypt depth (CD) were performed using a linear ocular micrometer (Olympus, ref. 209-35040, Microplanet, Barcelona, Spain) and the VH/CD ratio was calculated.

An additional sample of mid-jejunal mucosa (from a segment proximal to that used for morphometry) was obtained from the same birds (12 chicks per treatment). Total RNA was extracted with a RNeasy Mini Kit (QIAGEN, Hilden, Germany) following the manufacturer’s instructions. Contaminating DNA was digested using the RNase-free DNase Set (QIAGEN). The resulting mRNA was eluted in 50 µL of RNase-free water per 20 mg tissue and stored at −80 °C. The mRNA quantity and quality were determined by spectral analysis (BioPhotometer, Eppendorf, Hamburg, Germany), and only samples with an mRNA purity of about 2 (ratio E260/280) or above 2 (E260/230) were used. The expression rates of avian cytokines Interferon (IFN)-γ, IL-2, IL-10, Tumor necrosis factor (TNF)-α, and the cellular surface markers Toll-like receptor (TLR)-2, Major histocompatibility complex (MHC)-β-1 domain (MHC-I), and MHC class II glycoprotein (MHC-II), together with the Glyceraldehyde 3-phosphate dehydrogenase (GAPDH) reference gene, were determined using the QuantiTect SYBR^®^ Green RT-PCR Kit ref. 204243 (Qiagen GmbH, Hilden, Germany), according to the instructions of the manufacturer. Amplification and detection of specific products were performed on a 7500-Fast RT-PCR equipment (Applied Biosystems, - Thermo Fisher Scientific, Alcobendas, Spain) using the following temperature–time profile: one cycle of 50 °C for 30 min, 95 °C for 15 min, 45 cycles of 94 °C for 30 s, and 60 °C annealing temperature for 30 s followed by 72 °C for 30 s. A dissociation curve was drawn for each primer pair to assess the specificity of amplification products. Primer sequences and annealing temperatures are given in supplementary Table S1. Data were analyzed, applying the 2^−ΔΔCT^ method [18] for relative quantification and using the non-treated control group as calibrator.

### 2.7. Statistical Analysis

The pen was considered as the experimental unit for the variables measured in chickens that were allocated in pens, whereas the individual bird was considered an experimental unit for all the variables that were measured in chickens that were allocated in individual cages.

Performance, nutrient digestibility and excretion, bone mineralization, morphometry of intestinal mucosa, *SLC34A2* mRNA expression, and immunological variables were analyzed according to a randomized complete block design using the MIXED procedure of the SAS^®^ System for Windows V.9.4 (SAS Institute INC., Cary, NC, USA). The model included block (location in the experimental room) as a randomized effect, and CON, PL, and their interaction as main effects. The data in the tables are presented as least-square means and Fisher’s least significant difference method was applied to perform multiple comparisons. For gait score, FREQ and GLIMMIX procedures were applied, considering the main effects CON, PL, and their interaction and pen as random effect. Significant differences were declared at *p* ≤ 0.05, while 0.05 < *p* ≤ 0.10 were considered as tendencies.

## 3. Results

### 3.1. Performance

The performance results of the chickens that were allocated in pens are shown in Table 3. Significant effects of CON were observed during the first two weeks of age, as the conditioned chicks had reduced BW, ADG, and ADFI (all *p* < 0.01), although no effect was observed on FCR. During the third week of the trial (days 14 to 21), conditioned chickens still tended to have a lighter final BW (*p* = 0.098) but their FCR was significantly improved (*p* = 0.003). During the finisher phase (days 21 to 42) and over the whole trial (days 1 to 42), CON did not have any significant effect on performance. Conversely, there were significant effects of PL during these periods, as P-def resulted in reduced BW, ADG, and ADFI, and impaired the FCR of birds between days 21 to 42 (all *p* < 0.001) and the overall trial (all *p* < 0.01). There were no significant interactions between CON and PL for any of the performance measurements considered between days 21–42 or 1–42. However, it is worth noticing that during these periods, the conditioned/P-bal chickens had numerically improved ADG and FCR compared with chickens from the control/P-bal group.

### 3.2. Apparent Prececal Digestibility, Bone Mineralization, and Gait Score

Contradictory effects of CON on P-APCD were observed between the birds kept in pens (Table 4), and those that were assessed individually (Table 5). Whereas the conditioned chicks in pens tended to have improved P-APCD at day 43 compared to the control ones (62.0 vs. 57.8%; *p* = 0.100), the conditioned chicks that were kept individually had significantly worse P-APCD values at day 30 (41.1 vs. 48.9%; *p* = 0.023). Significant effects of PL were also observed at days 30 (individual) and 43 (pens), as the P-def diet presented lower P-APCD values than the P-bal diet (34.8 vs. 55.2%; *p* < 0.001 and 57.4 vs. 62.4%; *p* = 0.053, respectively). No significant interactions between CON and PL during the finisher phase were observed for P-APCD.

Tendencies to significant effects of CON on bone mineralization were observed at day 43 in the birds kept in pens (Table 4). The conditioned chicks tended to have increased tibia fresh weight (from 13.8 to 14.7 g; *p* = 0.073), tibia dry weight (from 6.51 to 6.89 g; *p* = 0.077), and tibia ash weight (from 2.598 to 2.753 g; *p* = 0.086), while no effects were observed on the percentages of tibia ash and dry matter percentages. Furthermore, PL had highly significant effects on all bone mineralization parameters, as the P-def diet reduced tibia fresh weight (from 14.9 to 13.5 g; *p* = 0.011), dry matter percentage (from 50.6 to 43.3%; *p* < 0.001), dry weight (from 7.53 to 5.86 g; *p* < 0.001), ash percentage (from 43.4 to 35.7%; *p* < 0.001), and ash weight (from 3.26 to 2.09 g; *p* < 0.001). No significant CON × PL interactions were observed for tibia mineralization traits at 43 d of age. On the other hand, for the individually kept birds, there were significant CON × PL interactions for tibia fresh and dry weights, and ash weight (*p* ≤ 0.05), and a tendency for ash percentage (*p* = 0.086) at 30 d of age (Table 5). For the conditioned chicks, the P-def treatment resulted in lower tibia fresh and dry weights and ash weight than the P-bal diet, whereas no differences between the two diets were found for the control chicks. A significant effect of PL on dry matter content was also observed; the tibias of birds that were fed the P-bal diet had a higher dry matter content that those that were fed the P-def diet (from 46.2 to 44.7%; *p* = 0.045).

Regarding the gait score assessment of the birds kept in pens (Table 4), there was a significant effect of PL; the P-def diet significantly reduced the percentage of birds with gait scores 0 to1 and increased that of birds with scores 4 to 5 (*p* < 0.001). No significant effects of CON or CON × PL interactions were observed.

### 3.3. Apparent Total Tract Digestibility and Nutrient Excretion

The effects of CON and PL on nutrient ATTD and excretion are shown in Table 6. Between days 19 to 21, a significant effect of CON on the relative excretion of phosphorous (expressed per g of weight gain) was observed, as the relative excretion of the conditioned chicks was lower than that of the control ones (4.16 vs. 4.41 mg/g gain; *p* = 0.045). No significant effects of CON on nutrient ATTD or any of the other variables analyzed were observed for this period. Between days 27 to 29 d, there were no significant effects of CON for nutrient ATTD or excretion. Statistically significant effects were observed for PL; relative to P-bal diet, the P-def diet resulted in a lower ATTD of phosphorous (49.5 vs. 42.6%; *p* < 0.001), lower phosphorous content in excreta (10.88 vs. 6.08 g/kg; *p* < 0.001), and lower phosphorous excretion per bird (327 vs. 178 mg/bird/d; *p* < 0.001) or per g of gain (4.70 vs. 2.78 mg/g gain; *p* < 0.001). On the contrary, P-def increased N concentration in excreta (36.8 vs. 38.5 g/kg; *p* = 0.035) and the relative amount of N excreted per g of gain (8.37 vs. 9.30 mg/g gain; *p* = 0.006). No CON × PL interactions for nutrient ATTD or P and N excretion were observed.

### 3.4. Gut Expression of SLC34A2

The expression of *SLC34A2* was greater in the epithelium of the duodenum than in that of the jejunum (19.5% more mRNA expression; *p* < 0.05). Conditioning had no significant effect on *SLC34A2* expression at day 22 d in either segment (Table 7). However, at 30 d, significant effects for both CON (*P* = 0.025) and PL (*p* < 0.001) were observed. Relative to the control, the conditioned chicks had increased *SLC34A2* expression in the duodenum by 1.18-fold, and relative to P-bal, the birds on P-def increased it by 1.30-fold. Conversely, in jejunum, only PL had a significant effect on *SLC34A2* expression, which was up-regulated 1.76-fold with P-def (*p* < 0.001). No CON × PL interactions were found.

### 3.5. Histology and Immunological Traits of Broiler Chickens

The effects of CON and PL on the morphometry of intestinal mucosa in the mid-jejunum are shown in Table 8. Conditioning had no effect on villus height or crypt depth at either 22 or 30 d of age. However, PL had a significant effect on crypt depth at 30 d of age, as the crypts of birds on P-bal were significantly deeper than those of P-def birds (179 vs. 152 µm; *p* = 0.038). No significant CON × PL interactions were found.

The effects of CON and PL on the expression of pro- and anti-inflammatory cytokines and cellular surface markers in the mucosa of mid-jejunum are shown in Table 9. No statistically significant effects of CON, PL, or interactions between them were observed for any parameter at either 22 or 30 d of experiment.

## 4. Discussion

In this study, the effects of phosphorous nutritional conditioning during the first week of life on subsequent performance, nutrient digestibility, bone mineralization, gait score, intestinal morphometry, *SLC34A2* cotransporter, and immunological traits of chickens were evaluated. There is evidence that birds under phosphorous restriction respond with an adaptive process by increasing the expression of genes that encode intestinal P and Ca transporters [5,6] or by modulating mineral homeostasis through endocrine regulators (e.g., calcitriol and parathyroid hormone) [7]. It is well known that severe phosphorous dietary reductions of more than 35% combined [19] or not [20] with Ca reduction impair the performance of growing chickens. The current study assessed whether the nutritional conditioning of birds with low phosphorous diets during the first week of life improved their ability to use dietary phosphorous later in life, as suggested by Rousseau et al. [6] and Ashwell and Angel [21]. This strategy was evaluated under conditions of adequate or deficient phosphorous supply by using P-balanced or P-deficient diets during the finisher phase. In the present study, phosphorous conditioning was induced with reductions of 53% in non-phytate P and 34% of Ca during the first week of life and resulted in reduced BW, ADG, and ADFI during the first and second week of trial, without impairment of FCR. These effects during the early growing stages agree with those of Imari et al. [22] who applied reductions in Ca and available P of 30% between days 1 to 10 of age. Similar results were obtained by Javadi et al. [23], with 25 and 15% reductions in Ca and non-phytate P, respectively, up to day 21 of life, and by Omotoso et al. [7], with a 50% non-phytate P reduction up to 17 d of age. The latter study also observed that the birds that were fed the phosphorous-limiting diets increased the levels of calcitriol in serum and suggested endocrine control as a potential mechanism to maintain broiler mineral homeostasis. Under the conditions of the current trial, CON had no significant effect on performance when considering the overall experimental period (1 to 43 d), and contrary to what was expected, conditioning did not result in any improvement in performance when the chicks were offered the P-def diet during the finisher phase. On the other hand, feeding P-def diets in the finisher phase resulted in reduced BW, ADG, and ADFI and impaired FCR, confirming that the dietary phosphorous levels in the P-def diet were below the chicken’s nutritional requirements. Thus, unlike what was expected, phosphorous conditioning was not able to compensate for the dietary phosphorous deficiency applied during the finisher period. This contrasts with the results of Angel and Ashwell [2], who observed that chickens conditioned with diets with a 50% reduction in Ca and non-phytate P during the first 90 h of life were able to compensate for reductions of 60% in non-phytate P and 43% in Ca during the finisher phase. 

Compared to control birds, those that had been conditioned excreted 5.7% less phosphorous per unit of weight gain between days 19 to 21, while being fed identical and well-balanced diets. This suggests a better efficiency of phosphorous utilization in these birds because of conditioning. Larger improvements of up to 16.8% in phosphorous retention efficiency in chickens have also been observed with dietary Ca and non-phytate P reductions of around 20% between 0 and 8 days (conditioning) and during the finisher phase of 19 to 33 d of age [3], but contrary to our study, phosphorous retention was evaluated between 0 and 33 d of age, including periods during which diets with different phosphorous contents were used. In addition, Angel and Ashwell [2] did not observe any effect of conditioning (applied in the first 90 h of life) on the apparent absorption of phosphorous in birds that were fed balanced diets between 8 and 22 d of age. Conversely, we also found that conditioning in the first week of life reduced P-APCD at 30 d of life by 16%, which suggests that it does not offer any advantage for phosphorous utilization at that stage. It is possible that the effects of conditioning wear off after a few weeks. Regarding the effects of feeding diets deficient in Ca and non-phytate P during the finisher phase on Ca and P utilization, we observed reductions of more than 40% in the excretion of phosphorous between 27 and 29 d of age. This was expected and agrees with previous studies such as that by Javadi et al. [23], who reported reductions in Ca and P excretion of over 30% when broilers were fed diets deficient in Ca and non-phytate P during all their productive life. The apparent contradiction between the performance and the phosphorous utilization results in our study may be explained by the severe phosphorous deficiency that was applied, which cannot be counteracted by conditioning. It is possible that using less severe phosphorous reductions may have resulted in performance improvements, but this is yet to be confirmed.

In our study, dietary non-phytate P restriction during the finisher phase reduced phosphorous ATTD by 14% between 27 and 29 d of age, and reduced (by 37.0%) or tended to decrease (by 8.10%) the P-APCD at 30 and 43 d of age, respectively. This is in contrast with the higher phosphorous retention efficiency that was observed by Imari et al. [22] between 11 and 42 d of life in chickens that were fed diets with 30% reductions in Ca and non-phytate P. Rousseau et al. [6] also found that reducing the levels of non-phytate P by reducing inorganic phosphates between days 10 to 21 resulted in improved Ca and P-APCD in chickens at 21 d of life. The lower APCD and ATTD of Ca and P for the non-phytate P-deficient diets in our study were to be expected, as the reductions were achieved by removing inorganic phosphate (reduced by 74 and 98% in the starter and finisher diets, respectively), while the amounts of phytate-bound P were maintained. Our observations that both conditioning and feeding phosphorous-deficient diets reduced P-APCD at d 30, in contrast with the simultaneous increases in *SLC34A2* gene expression caused by conditioning in the duodenum and by phosphorous-deficient diets in the duodenum and jejunum. The conditioning effect on *SLC34A2* gene expression at day 30 but not day 22 suggests that the effects of conditioning may be long term. Indeed, other experiments demonstrated that newly hatched chickens challenged with a low phosphorous diet during the first 4 d of life or during the finisher phase (22 to 38 d) increased expression of the specific Na-P co-transporter *SLC34A2* in the intestine at 4 or 38 d, respectively [21]. Unlike in our study, these increases in gene expression were accompanied by improvements in the utilization of dietary P. It has been shown that feeding low phosphorous diets to conditioned birds increased the expression of phosphorous transporters *SLC20A1* and *SLC34A2*, and that they coincided with an enhancement of phosphorous digestibility, which confirmed a transcriptional regulation of phosphorous efficiency [6]. Other studies failed to demonstrate an effect of reduced dietary levels of phosphorous on the expression of *SLC34A2* throughout the grower/finisher phase [3,7]. The current results show that *SLC34A2* expression is highly regulated in the duodenum compared with the jejunum in chickens after phosphorous conditioning during the first week of life, which is in agreement with Yan et al. [5]. Fang et al. [9] suggested different regulatory mechanisms in these tissues; the duodenum seems to play the main role in the chronic adaptation to a low-inorganic-phosphorous diet, while the jejunum plays a major role in the acute response to a high-inorganic-phosphorous diet. However, in our study, *SLC34A2* gene expression was increased in both the duodenum and jejunum during phosphorous restriction in the finisher phase. Taken together, the current results indicate that conditioning during the first week of life and phosphorous deficiency in the finisher phase increased *SLC34A2* gene expression in the duodenum at 30 d, although this increase was not enough to counteract the negative effects of P-def on performance and bone mineralization. It is possible that the birds could not take full advantage of this adaptation as most of the non-absorbed phosphorous may have been in phytate form. From a practical point of view, the combination of this strategy with the use of phytases may be advantageous and needs to be validated. 

The continuous improvements in the growth rate of commercial breeds of broiler chickens, combined with the reduced levels of dietary phosphorous for environmental purposes, have increased the risk of deficient bone mineralization, which can lead to leg weakness and impaired walking ability that can compromise the welfare of the birds. We observed significant interactions between CON and PL for tibia mineralization at day 30, whereas conditioning during the first week of age improved bone mineralization in the birds that were fed the phosphorous-balanced diet during the finisher phase, and it had the opposite effect on the birds that were fed the phosphorous-deficient diet. At day 43, such an interaction was no longer observed and despite the phosphorous-deficient diet in the finisher phase having a negative effect on bone mineralization and gait score, conditioning during the first week of life still improved bone mineralization. In a study by Rousseau et al. [6], bone mineralization was also impaired at 35 d by dietary phosphorous deficiency between days 10 to 21, but only when Ca levels had not been simultaneously reduced. However, the same study showed that birds fed Ca- and phosphorous-deficient diets between days 10 to 21 followed by a balanced diet between 22 and 35 d tended to have increased tibia ash weight and bone breaking strength at 35 d, compared to birds that had always been feed according to requirements. Other studies have also reported reduced bone mineralization in conditioned birds (between 0 and 8 d of age) that were fed a low non-phytate P diet between 19 and 33 d [3]. However, in the same study, the bone mineralization of birds that had been conditioned for a shorter period of time (between 0 and 4 d of age) did not differ from that of the control birds that had been fed adequate diets throughout their life. This suggests that the length of the conditioning period may be important when designing such a strategy. Dietary reductions of 30% in Ca and non-phytate P between 0 and 42 d have been reported to reduce the contents of ash, Ca, and P in the tibia of chickens at 42 d of age [22]. Similarly, impaired bone mineralization was also observed in chickens fed grower and finisher diets with reductions of around 30 and 70% for non-phytate P and Ca, respectively [24], 35% for non-phytate P [20], or 49% for non-phytate P and 65% for Ca [19]. In the latter study, the impairment of bone mineralization was more severe when phosphorous deficiency was combined with adequate levels of Ca. However, the phosphorous content in tibia ash of birds that were fed diets deficient in Ca and P was even higher than that observed in birds fed adequate diets.

The inflammatory response induced under conditions of stress or pathogenic infections changes the partitioning of nutrients away from body protein accretion, thereby reducing the growth potential of the chicks [13,25]. Hence, phosphorous deficiency may not only impair growth performance and bone development but may also modify the immune response of birds. In the current study, the reduced dietary non-phytate P levels associated with conditioning during the first week of life or feeding phosphorous-deficient diet during the finisher phase did not modify the expression of immune signaling molecules such as interleukins or cellular receptors in the jejunum mucosa. This suggests that these interventions did not induce an inflammatory process. Although there is little information on the effects of reducing the dietary levels of Ca and P on the immune response in the gastrointestinal tract, a recent study has reported that dietary Ca and P reduction enhanced cytokine expression in the spleen, with an IL-12/IL-10 ratio that suggested an anti-inflammatory status [11]. 

## 5. Conclusions

Taken together, the results of this study indicate that conditioning with a deficiency of non-phytate P during the first week post-hatching did not affect the overall performance at the end of the experiment. Nonetheless, we observed that conditioning reduced the excretion of phosphorous per unit of weight gain shortly after conditioning, and at later stages, it significantly increased the duodenal gene expression of *SLC34A2* and tended to improve the digestibility of phosphorous and tibia mineralization. However, these effects were not able to counteract the adverse effects of phosphorous deficiency during the finishing phase on growth, bone mineralization, and gait score at the end of the trial. From a practical point of view, the conditioning procedure that we used may need to be revised by using less severe P-reductions and/or the simultaneous use of phytases. If a successful strategy is achieved, this could be easily used independently of farm size and rearing system, offering great potential to reduce phosphorous excretion and the dependency on the use of non-renewable sources of inorganic P. 

It is concluded that phosphorous nutritional conditioning during the first days post-hatch may still be seen as an opportunity to increase dietary phosphorous utilization in poultry, but additional research is required to optimize the duration and extent of the conditioning protocol, as well as the magnitude of phosphorous reduction during the post conditioning periods.

## Figures and Tables

**Table 1:** Ca and P levels in experimental diets.

Treatments		Starter Diet (1–7 d)	Grower Diet (7–21 d)	Finisher Diet (21–43 d)
Conditioning (CON)	P level in Finisher Diet (PL)			
Control	P-bal	9.0 g/kg Ca 4.5 g/kg nPP * 6.9 g/kg total P	8.4 g/kg Ca 4.2 g/kg nPP 6.5 g/kg total P	7.6 g/kg Ca 3.5 g/kg nPP 5.8 g/kg total P
	P-def			4.5 g/kg Ca 1.2 g/kg nPP 3.5 g/kg total P
Conditioned	P-bal	5.9 g/kg Ca 2.1 g/kg nPP 4.5 g/kg total P		7.6 g/kg Ca 3.5 g/kg nPP 5.8 g/kg total P
	P-def			4.5 g/kg Ca 1.2 g/kg nPP g/kg total P

* nPP: non-phytate phosphorus.

**Table 2:** Ingredient composition and nutrient content of diets (g/kg as-fed).

	Starter (1–7 d)		Grower (7–21 d)	Finisher (21–21 d)	
	Control	Conditioning	Control	Balanced	Deficient
Ingredients					
Maize	582	606	630	653	676
Soybean meal 48% CP	342	338	293	272	268
Soybean oil	37.2	29.8	40.5	44.3	36.9
Calcium carbonate	6.30	7.41	5.99	6.46	7.47
Dicalcium phosphate	18.7	4.94	17.4	13.8	0.25
Sodium chloride	3.78	3.79	3.80	3.56	3.58
L-lysine HCl	2.48	2.55	2.50	1.46	1.54
DL-methionine	3.33	3.32	2.84	2.46	2.45
L-threonine	0.73	0.73	0.70	0.33	0.33
Premix *	3.00	3.00	3.00	3.00	3.00
Ethoxyquin 66% ^†^	0.20	0.20	0.20	0.20	0.20
Calculated nutrient content					
AMEn (kcal/kg)	3035	3035	3100	3150	3150
Crude protein (g/kg)	210	210	190	180	180
Lysine (g/kg)	13.2	13.2	11.9	10.5	10.5
Methionine (g/kg)	6.46	6.46	5.73	5.24	5.24
Methionine + cystine (g/kg)	9.89	9.90	8.90	8.31	8.32
Threonine (g/kg)	8.60	8.60	7.80	7.10	7.10
Tryptophan (g/kg)	2.37	2.35	2.09	1.97	1.95
Calcium (g/kg)	9.00	5.90	8.40	7.60	4.51
Total phosphorus (g/kg)	6.91	4.54	6.50	5.80	3.47
Non-phytate phosphorus (g/kg)	4.50	2.10	4.18	3.52	1.15
Calcium /total P	1.30	1.30	1.29	1.31	1.30
Analyzed nutrient content					
Gross energy (kcal/kg)	4060	4086	4071	4096	4112
Dry matter (g/kg)	858	856	867	871	863
Crude protein (g/kg)	205	201	183	170	175
Ether extract (g/kg)	56.9	53.6	62.5	67.5	61.0
Ash (g/kg)	50.8	43.1	43.0	50.2	41.6
Calcium (g/kg)	10.0	7.0	8.5	8.0	5.0
Phosphorus (g/kg)	6.6	4.6	6.0	5.4	2.6

* The premix provided per kg feed: vitamin A (E672) 13500 UI; vitamin D_3_ (E 671) 4800 UI; vitamin E (α-tocopherol) 67.2 IU; vitamin B_1_ 3 mg; vitamin B_2_ 9 mg; vitamin B_6_ 4.5 mg; vitamin B_12_ 16.5 µg; vitamin K_3_ 3 mg; calcium panthotenate 16.5 mg; nicotinic acid 51 mg; folic acid 1.8 mg; biotin 30 µg; Fe (E1) (from FeSO_4_•7H_2_O) 54 mg; I (E2) (from Ca(I_2_O_3_)_2_) 1.2 mg; Co (E3) (from 2CoCO_3_·3Co(OH)_2_·H2O) 0.6 mg; Cu (E4) (from CuSO_4_·5H_2_O) 12 mg; Mn (E5) (from MnO) 90 mg; Zn (E6) (from ZnO) 66 mg; Se (E8) (from Na_2_SeO_3_) 0.18 mg; Mo (E7) ((NH_4_)_6_Mo_7_O_24_) 1.2 mg. ^†^ Providing 133 mg ethoxyquin/kg of feed.

**Table 3:** Effects of phosphorous nutritional conditioning (CON) and phosphorous level during the finisher phase (PL) on performance of broiler chickens allocated in pens ^1^.

CON:	Control		Conditioned		*SE*	*p*-Value		
PL:	P-bal	P-def	P-bal	P-def		CON	PL	CON × PL
BW (g)								
7 d	182		176		3.13	0.006	-	-
14 d	463		442		6.41	<0.001	-	-
21 d	895		876		14.9	0.098	-	-
42 d	3038	2724	3084	2736	60.7	0.543	<0.001	0.710
ADG (g/d)								
1–7 d	19.6		18.7		0.45	0.006	-	-
7–14 d	40.1		38.1		0.70	0.001	-	-
14–21 d	61.8		62.0		1.28	0.905	-	-
21–42 d	101.7	87.4	105.2	88.4	2.17	0.283	<0.001	0.537
1–42 d	71.2	63.8	72.4	64.1	1.45	0.541	<0.001	0.708
ADFI (g/d)								
1–7 d	22.9		21.9		0.40	0.002	-	-
7–14 d	60.3		56.6		0.89	<0.001	-	-
14–21 d	96.1		93.6		1.54	0.128	-	-
21–42 d	181.6	162.8	184.2	164.6	3.23	0.441	<0.001	0.888
1–42 d	120.9	110.4	120.7	111.0	2.03	0.895	<0.001	0.766
Feed Conversion Ratio (g/g)								
1–7 d	1.165		1.175		0.0124	0.525	-	-
7–14 d	1.504		1.487		0.0121	0.273	-	-
14–21 d	1.557		1.511		0.0151	0.003	-	-
21–42 d	1.785	1.863	1.750	1.866	0.0166	0.362	<0.001	0.283
1–42 d	1.696	1.730	1.667	1.735	0.0121	0.337	0.002	0.186

P-bal: phosphorous-balanced finisher diet; P-def: phosphorous-deficient finisher diet. ^1^ Values are least-square means. *n* = 12 between 1–7 and 7–14 d of age. *n* = 10 between 14 and 21 d of age. *n* = 4 between 21–42 and 1–42 d of age. Each replicate consisted of 25 chickens per pen.

**Table 4:** Effects of phosphorus nutritional conditioning (CON) and phosphorous level during the finisher phase (PL) on phosphorous-apparent prececal digestibility (APCD), tibia mineralization, and gait score at 43 d of broiler chickens allocated in pens ^1^.

CON:	Control		Conditioned		*SE*	*p*-Value		
PL:	P-bal	P-def	P-bal	P-def		CON	PL	CON × PL
P-APCD (%) ^2^	59.9	55.7	64.9	59.0	2.46	0.100	0.053	0.729
Tibia mineralization ^3^								
Fresh weight (g)	14.1	13.5	15.8	13.6	0.73	0.073	0.011	0.104
Dry matter (%)	51.2	43.1	50.0	43.6	0.70	0.564	<0.001	0.222
Dry weight (g)	7.21	5.80	7.85	5.93	0.350	0.077	<0.001	0.229
Ash percentage (%)	43.4	36.0	43.5	35.4	0.78	0.685	<0.001	0.584
Ash weight (g)	3.12	2.08	3.41	2.10	0.137	0.086	<0.001	0.138
Gait score ^4^								
Score 0–1 (%)	9.82 ^a^	4.46 ^b^	10.7 ^a^	1.79 ^c^	-			
Score 2–3 (%)	14.3 ^a^	15.2 ^a^	13.4 ^a^	17.0 ^a^	-	0.659	<0.001	0.567
Score 4–5 (%)	0.89 ^b^	5.36 ^a^	0.89 ^b^	6.25 ^a^	-			

P-bal: phosphorous-balanced finisher diet; P-def: phosphorous-deficient finisher diet. ^1^ Values are least-square means. *n* = 4. ^2^ Each replicate consisted of 3 composite samples (3 birds) per pen. ^3^ Each replicate consisted of 3 birds per pen. ^4^ Each replicate consisted of 7 birds per pen. ^abc^ Values in the same row with different letters are significantly different (*p* < 0.05).

**Table 5:** Effects of phosphorus nutritional conditioning (CON) and phosphorous level during the finisher phase (PL) on phosphorous-apparent prececal digestibility (APCD) and tibia mineralization at 30 d of broiler chickens individually allocated in cages ^1^.

CON:	Control		Conditioned		*SE*	*p*-Value		
PL:	P-bal	P-def	P-bal	P-def		CON	PL	CON × PL
P-APCD (%)	58.8	38.9	51.5	30.6	3.34	0.023	<0.001	0.873
Tibia mineralization								
Fresh weight (g)	6.88 ^ab^	7.01 ^a^	7.21 ^a^	6.55 ^b^	0.190	0.720	0.181	0.050
Dry matter (%)	46.4	45.0	45.9	44.3	0.67	0.429	0.045	0.912
Dry weight (g)	3.19 ^a^	3.14 ^a^	3.30 ^a^	2.90 ^b^	0.082	0.438	0.012	0.039
Ash percentage (%)	41.3	38.4	43.4	38.0	0.69	0.263	<0.001	0.086
Ash weight (g)	1.32 ^ab^	1.21 ^bc^	1.43 ^a^	1.10 ^c^	0.393	0.960	<0.001	0.010

P-bal: phosphorous-balanced finisher diet; P-def: phosphorous-deficient finisher diet. ^1^ Values are least-square means. *n* = 12. ^abc^ Values in the same row with different letters are significantly different (*p* < 0.05).

**Table 6:** Effects of phosphorus nutritional conditioning (CON) and phosphorous level during the finisher phase (PL) on apparent total tract digestibility (ATTD) and nutrient excretion of broiler chickens individually allocated in cages ^1^.

CON:	Control		Conditioned		*SE*	*p*-Value		
PL:	P-bal	P-def	P-bal	P-def		CON	PL	CON × PL
ATTD and nutrient excretion between 19 and 21 d of age								
Dry matter ATTD (%)	73.2		73.1		0.23	0.675	-	-
Energy ATTD (%)	77.9		77.8		0.27	0.649	-	-
Phosphorus ATTD (%)	49.9		51.3		0.92	0.161	-	-
Nitrogen ATTD (%)	84.2		84.6		0.31	0.351	-	-
P in excreta (g/kg DM)	11.8		11.4		0.23	0.123	-	-
N in excreta (g/kg DM)	34.6		34.4		0.36	0.685	-	-
P excretion (mg/bird/d)	236		235		6.5	0.928	-	-
P excretion (mg/g gain)	4.41		4.16		0.104	0.045	-	-
N excretion (mg/bird/d)	362		362		9.5	0.997	-	-
N excretion (mg/g gain)	6.78		6.42		0.172	0.138	-	-
ATTD and nutrient excretion between 27 and 29 d of age								
Dry matter ATTD (%)	73.4	74.0	73.6	73.2	0.43	0.497	0.834	0.279
Energy ATTD (%)	78.3	79.0	78.6	78.3	0.58	0.754	0.679	0.341
Phosphorus ATTD (%)	49.6	41.9	49.3	43.2	1.32	0.688	<0.001	0.540
Nitrogen ATTD (%)	82.5	82.3	82.3	81.9	0.56	0.624	0.619	0.845
P in excreta (g/kg DM)	10.92	6.25	10.83	5.91	0.229	0.356	<0.001	0.600
N in excreta (g/kg DM)	36.6	38.0	37.1	38.9	0.95	0.397	0.035	0.788
P excretion (mg/bird/d)	329	185	325	170	9.7	0.344	<0.001	0.563
P excretion (mg/g gain)	4.68	2.75	4.72	2.81	0.124	0.707	<0.001	0.927
N excretion (mg/bird/d)	577	606	590	586	27.1	0.916	0.649	0.549
N excretion (mg/g gain)	8.20	9.01	8.54	9.59	0.326	0.167	0.006	0.725

P-bal: phosphorous-balanced finisher diet; P-def: phosphorous-deficient finisher diet. ^1^ Values are least-square means. *n* = 48 for 19–21 d of age. *n* = 24 for 27–29 d of age.

**Table 7:** Effects of phosphorus nutritional conditioning (CON) and phosphorous level during the finisher phase (PL) on the Type IIb Sodium Phosphate Cotransporter (*SLC34A2*) gene expression in duodenum and jejunum of broiler chickens individually allocated in cages at 22 and 30 d ^1^.

CON:	Control		Conditioned		*SE*	*p*-Value		
PL:	P-bal	P-def	P-bal	P-def		CON	PL	CON × PL
At 22 d (2^−ΔΔC^_T_)								
Duodenum	1.00		0.88		0.017	0.267	-	-
Jejunum	1.00		0.92		0.020	0.601	-	-
At 30 d (2^−ΔΔC^_T_)								
Duodenum	1.00	1.26	1.14	1.51	0.018	0.025	<0.001	0.471
Jejunum	1.00	2.03	1.04	1.56	0.025	0.279	<0.001	0.214

P-bal: phosphorous-balanced finisher diet; P-def: phosphorous-deficient finisher diet. ^1^ Values are least-square means. *n* = 12.

**Table 8:** Effects of phosphorus nutritional conditioning (CON) and phosphorous level during the finisher phase (PL) on the mid-jejunum morphometry of broiler chickens individually allocated in cage ^1^.

CON:	Control		Conditioned		*SE*	*p*-Value		
PL:	P-bal	P-def	P-bal	P-def		CON	PL	CON × PL
At 22 d of age								
Villus height (µm)	774		754		43.2	0.750	-	-
Crypt depth (µm)	203		204		12.2	0.977	-	-
Villus/crypt ratio	4.05		3.84		0.25	0.556	-	-
At 30 d of age								
Villus height (µm)	863	849	878	829	49.9	0.964	0.535	0.730
Crypt depth (µm)	188	147	170	156	12.6	0.742	0.038	0.309
Villus/crypt ratio	4.98	6.22	5.51	5.82	0.51	0.890	0.137	0.367

P-bal: phosphorous-balanced finisher diet; P-def: phosphorous-deficient finisher diet. ^1^ Values are least-square means. *n* = 12 for 22 d of age. *n* = 8 for 30 d of age.

**Table 9:** Effects of phosphorus nutritional conditioning (CON) and phosphorous level during the finisher phase (PL) on immunological traits of the mid-jejunum mucosa of broiler chickens individually allocated in cages ^1^.

CON:	Control		Conditioned		*SE*	*p*-Value		
PL:	P-bal	P-def	P-bal	P-def		CON	PL	CON × PL
At 22 d of age								
INF-γ (2^−ΔΔC^_T_)	1.00		1.09		0.142	0.524	-	-
IL-2 (2^−ΔΔC^_T_)	1.00		0.82		0.410	0.635	-	-
IL-10 (2^−ΔΔC^_T_)	1.00		0.93		0.142	0.613	-	-
TNF-α (2^−ΔΔC^_T_)	1.00		0.98		0.234	0.931	-	-
TLR-2 (2^−ΔΔC^_T_)	1.00		1.03		0.138	0.812	-	-
MHC I (2^−ΔΔC^_T_)	1.00		2.45		1.121	0.424	-	-
MHC II (2^−ΔΔC^_T_)	1.00		0.98		0.128	0.900	-	-
At 30 d of age (2^−ΔΔC^_T_)								
INF-γ (2^−ΔΔC^_T_)	1.00	1.17	0.95	1.07	0.134	0.437	0.129	0.853
IL-2 (2^−ΔΔC^_T_)	1.00	0.67	0.74	1.07	0.785	0.883	0.795	0.485
IL-10 (2^−ΔΔC^_T_)	1.00	0.88	0.40	0.71	0.623	0.285	0.218	0.158
TNF-α (2^−ΔΔC^_T_)	1.00	1.58	0.98	0.58	0.861	0.393	0.956	0.415
TLR-2 (2^−ΔΔC^_T_)	1.00	1.19	0.95	1.11	0.181	0.627	0.187	0.955
MHC I (2^−ΔΔC^_T_)	1.00	1.19	1.35	1.34	0.221	0.174	0.581	0.549
MHC II (2^−ΔΔC^_T_)	1.00	1.52	0.50	0.99	0.714	0.263	0.270	0.784

P-bal: phosphorous-balanced finisher diet; P-def: phosphorous-deficient finisher diet; INF-γ = Interferon-gamma; IL = Interleukin; MHC = Major histocompatibility complex; TLR = Toll-like receptor; TNF-α = Tumor necrosis factor alpha protein. ^1^ Values are least-square means. *n* = 12.

## Data Availability

The data presented in this study are available on request from the corresponding author.

## Supplementary Materials

The following supporting information can be downloaded at https://www.mdpi.com/article/10.3390/ani14223218/s1, Table S1: Primers used for the gene expression analysis of immune and SLC34A2 cotransporter genes in ileum.
