# Supplementary material for: Long-Term Effects of Early Low-Phosphorous Nutritional Conditioning on Broiler Chicken Performance, Bone Mineralization, and Gut Health Under Adequate or Phosphorous-Deficient Diets
Source: Animals (Basel). 2024 Nov 9;14(22):3218. doi: 10.3390/ani14223218 (PMC11591060; doi:10.3390/ani14223218)
Supplement: Supplementary file 1 [file animals-14-03218-s001.zip › animals-3261953-SI.pdf]

Impact of early low-phosphorous nutritional conditioning on subsequent performance, bone mineralization, and gut health in broiler chickens fed adequate or phosphorous-deficient diets

Núria Tous [1], Maria Francesch [1], Joan Tarradas [1], Ignacio Badiola [2], Ana M. Pérez de Rozas [2], Emma Fàbrega [3], Maria Ballester [4], Raquel Quintanilla [4], David Torrallardona [1,*]

[1] IRTA - Animal Nutrition, E-43120 Constantí, Spain; nuria.tous@irta.cat (N.T.); maria.francesch@irta.cat (M.F.); joan.tarradas@irta.cat (J.T.)
[2] IRTA - CReSA Animal Health, E-08193 Bellaterra, Spain; ignacio.badiola@irta.cat (I.B.); ana.perezderozas@irta.cat (A.M.P.R.)
[3] IRTA - Animal Welfare, E-17121 Monells, Spain; emma.fabrega@irta.cat
[4] IRTA - Animal Breeding and Genetics, E-08140 Caldes de Montbui, Spain; maria.ballester@irta.cat (M.B.); raquel.quintanilla@irta.cat (R.Q.)
* Correspondence: david.torrallardona@irta.cat

Citation: To be added by editorial staff during production.

Academic Editor: Firstname Lastname

Received: date
Revised: date
Accepted: date
Published: date

Copyright: © 2024 by the authors. Submitted for possible open access publication under the terms and conditions of the Creative Commons Attribution (CC BY) license (https://creativecommons.org/licenses/by/4.0/).

Supplementary Table S1. Primers used for the gene expression analysis of immune and *SLC34A2* cotransporter genes in ileum.

Gene	Forward sequence	Reverse sequence
Immune biomarkers		
IFN-γ	5′-CCCGATGAACGACTTGAGAAT-3′	5′-AGACTGGCTCCTTTTCCTTTTG-3′
IL-2	5′-CCCAGCAAACTCTGCAGTGTT-3′	5′-CCGGTGTGATTTAGACCCGTA-3′
IL-10	5′-CATGCTGCTGGGCCTGAA-3′	5′-CGTCTCCTTGATCTGCTTGATG-3′
TNF-α	5′-AAGCCAAGAGCACACCTGAC-3′	5′-CAGGTATCACCAGTGCGTTG-3′
TLR-2	5′-TTTGTGCCTGGGAAGTGGATT-3′	5′-ATGAGGATCGCCACATCG-3′
MHC I	5′-CAGGGAAGAAGGGGAAGG-3′	5′-GAAGCACAGCACTCAGATGG-3′
MHC II	5′-CACTACCTGAACGGCACC-3′	5′-AATCTCGGCGTTGCTGTTCC-3′
[1]*GAPDH*	5′-GGGTGTCAACCATGAGAAATAT-3′	5′-CCCTCCACAATGCCAAAGTT-3′
Co-transporter analysis		
SLC34A2	5′-TCTACTTGTTCGTGTGTTCTCTGGAT 3′	5′ CCCCTGCTGCTTTACCTCCTA-3′
[1]*B2M*	5′-GTGCTGGTGACCCTGGTG 3′	5′-CAGTTGAGGACGTTCTTGGTG-3′
[1]*GAPDH*	5′-AGCTGATGCCCCCATGTTT 3′	5′-GCACGATGCATTGCTGACA-3′

Interferon (IFN); Interleukin (IL), Tumor necrosis factor (TNF), Toll-like receptor (TLR), Major histocompatibility complex (MHC)-β-1 domain (MHC-I), and MHC class II glycoprotein (MHC-II), Glyceraldehyde 3-phosphate dehydrogenase (GAPDH), Type IIb Sodium Phosphate Co-transporter (SLC34A2), β2-microglobulin (B2M).

[1]Reference genes.
